# Case Report: A case of ruptured renal epithelioid angiomyolipoma leading to the diagnosis of TSC2/PKD1 contiguous gene syndrome

**DOI:** 10.3389/fped.2026.1756327

**Published:** 2026-02-06

**Authors:** Takato Akiba, Shino Shimada, Michiaki Ikegami, Naoto Nishizaki, Akane Hashizume, Taiji Nozaki, Yoji Nagashima, Akira Tsujimura, Nana Nakazawa-Tanaka, Go Miyano, Ken Takahashi, Hiromichi Shoji

**Affiliations:** 1 Department of Pediatrics, Juntendo University Urayasu Hospital, Urayasu, Japan; 2 Department of Pediatrics and Adolescent Medicine, Juntendo University Graduate School of Medicine, Tokyo, Japan; 3 Department of Pediatric Surgery, Juntendo University Urayasu Hospital, Urayasu, Japan; 4 Department of Pathology, Juntendo University Urayasu Hospital, Urayasu, Japan; 5 Department of Urology, Juntendo University Urayasu Hospital, Urayasu, Japan; 6 Department of Surgical Pathology, Tokyo Women’s Medical University Hospital, Tokyo, Japan

**Keywords:** chromosomal microarray analysis, epithelioid angiomyolipoma, polycystic kidney disease, renal hemorrhage, TSC2/PKD1 contiguous gene syndrome (PKDTS), tuberous sclerosis complex

## Abstract

**Background:**

Tuberous sclerosis complex (TSC) is frequently complicated by renal lesions, including angiomyolipoma (AML), renal cysts, and renal cell carcinoma (RCC). Large deletions involving adjacent *TSC2* and *PKD1* genes cause *TSC2*/*PKD1* contiguous gene syndrome (PKDTS), which carries a risk of early renal decline. Epithelioid AML (eAML), to the best of our knowledge, has not been previously reported in children with PKDTS.

**Case presentation:**

A 13-year-old boy with hypomelanotic macules and facial angiofibromas presented with acute abdominal pain and fever; CT revealed a ruptured heterogeneous 5-cm right renal cystic, and multiple cysts. Robot-assisted partial nephrectomy confirmed epithelioid angiomyolipoma (eAML) via pathology and immunohistochemistry (cathepsin K+, CD10/p53 partial+, others negative). Neuroimaging and ophthalmology revealed TSC features; chromosomal microarray identified an ∼882-kb 16p13.3 deletion encompassing *TSC2*/*PKD1*, diagnosing PKDTS.

**Conclusions:**

PKDTS may manifest in childhood as an eAML rupture. In pediatric TSC, eAML or RCC should not be excluded based on age. Atypical findings (e.g., calcification or necrosis) warrant early biopsy; non-diagnostic sequencing requires copy-number analysis (e.g., chromosomal microarray) to detect *TSC2* deletions in TSC-featured patients and multiple renal cysts.

## 1 Introduction

Tuberous sclerosis complex (TSC) is an autosomal dominant neurocutaneous disorder associated with pathogenic variants of *TSC1* or *TSC2*. *TSC2* is located on chromosome 16p13.3, adjacent to polycystin 1 (*PKD1*), pathogenic variants of which are responsible for autosomal dominant polycystic kidney disease (ADPKD) (1). Large chromosomal deletions encompassing *TSC2* and *PKD1* can lead to combined haploinsufficiency, resulting in *TSC2*/*PKD1* contiguous gene syndrome (PKDTS, OMIM #600273), which is characterized by overlapping clinical features of TSC and ADPKD (2).

Although the renal manifestations of TSC can differ, angiomyolipoma (AML), renal cysts, and renal cell carcinoma (RCC) are common features (3). Approximately 60% of patients with *TSC2* variants develop renal AML (4), of which, epithelioid angiomyolipoma (eAML), a rare histological subtype characterized by epithelioid morphology, is extremely uncommon in children (5). Although in a majority of cases, renal lesions in TSC remain asymptomatic, AMLs can enlarge rapidly, particularly during adolescence or early adulthood, thereby posing a risk of spontaneous rupture and life-threatening hemorrhage.

Herein, we describe the case of a 13-year-old male patient with genetically confirmed PKDTS, who presented with acute abdominal pain due to rupture of an eAML. Given that rupture of an eAML is a plausible presenting symptom owing to a risk of rapid AML enlargement in this population, this case highlights the need for vigilant surveillance and timely intervention, and emphasizes the importance of early detection and management of renal involvement in pediatric TSC for optimizing long-term patient outcomes (6, 7).

## 2 Case description

The patient was a 13-year-old male, the first of two siblings, with no notable medical or family history. At 3 years of age, he had experienced febrile status epilepticus, with brain magnetic resonance imaging (MRI) revealing cortical tubers, raising clinical suspicion of TSC. Although further outpatient evaluation was planned, follow-up was discontinued owing to the patient's anxiety regarding medical visits. However, on the day prior to admission, the patient had developed intermittent abdominal pain in the epigastric and right lower quadrants, and on presentation, he had developed a fever of 38 ℃, along with worsening tenderness and abdominal pain, prompting hospital admission.

**Figure 1:**
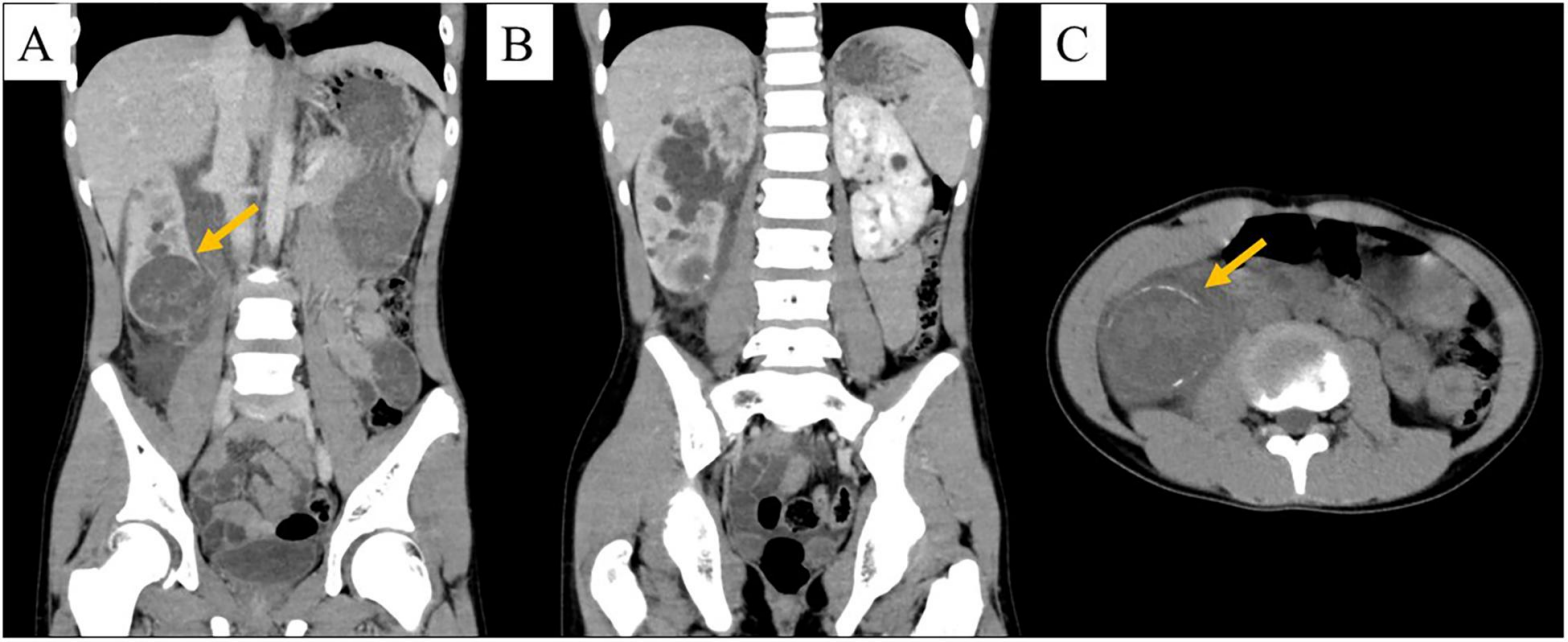
Abdominal computed tomography. **(A,B)** Contrast-enhanced. **(C)** unenhanced. **(A)** Heterogeneous ∼5-cm cystic lesion at the inferior pole of the right kidney (orange arrow) with focal wall discontinuity compatible with a rupture. **(B)** Numerous bilateral renal cysts, with slightly reduced enhancement of the right kidney compared with the left kidney (etiology undetermined). **(C)** The same cystic lesion with peripheral rim calcifications (orange arrow).

The vital signs were as follows: temperature, 38.0 ℃; heart rate, 96 bpm; blood pressure, 102/60 mmHg; and oxygen saturation, 98%. Multiple hypomelanotic macules were scattered over the trunk and extremities and facial angiofibromas were observed on both cheeks. Abdominal examination revealed tenderness from the right lower quadrant to the epigastrium, reduced bowel sounds, muscle guarding, and tenderness at the right costovertebral angle. Laboratory tests indicated leukocytosis (white blood cell count 17,600/μL; neutrophils 82.6%) without evidence of renal dysfunction, and contrast-enhanced computed tomography (CT) of the chest and abdomen revealed multiple bilateral renal cysts and a 5-cm cystic lesion with a heterogeneous internal density at the upper pole of the right kidney. The lesion was found to be characterized by focal wall disruption and peripheral calcification (Figure 1), and mild ascites were also noted.

On the basis of these findings, retroperitoneal inflammation secondary to rupture of a right renal cystic lesion was diagnosed. However, although the patient's condition improved with intravenous antibiotic therapy, given that the cystic lesion showed peripheral calcification, the possibility of malignancy, including renal cell carcinoma (RCC), could not be excluded, and, consequently, elective robot-assisted laparoscopic partial nephrectomy of the right kidney was performed (Table 1).

**Table 1:** Clinical timeline of key events.

Age/Date	Event	Detail of clinical course
3 years	Febrile status epilepticus	Clinical suspicion of TSC
3–13 years	Loss to follow-up	Due to patient anxiety regarding medical visits
Day −1	Onset of symptom	Intermittent abdominal pain.
Day 0	ER vist, Hospitalization	Detect bilateral renal cysts and ruptured eAML. (retroperitoneal inflammation secondary to lesion rupture.)
Day 0–11	Intravenous antibiotics	Clinical improvement with antibiotic therapy alone.
Day 16	Targeted sequencing (TSC1/TSC2)	No pathogenic variants detected (result reported at ∼2 months).
Day 19	Biopsy	RCC and eAML considered in the differential diagnosis.
Month 2	Elective robot-assisted laparoscopic partial nephrectomy	Based on the pathological findings, a definitive diagnosis of eAML.
Month 8	CMA	Definitive diagnosis of PKDTS.

TSC, tuberous sclerosis complex; ER, emergency room; eAML, Epithelioid Angiomyolipoma; RCC, renal cell carcinoma; CMA, chromosomal microarray analysis; PKDTS, TSC2/PKD1 contiguous gene syndrome.

**Figure 2:**
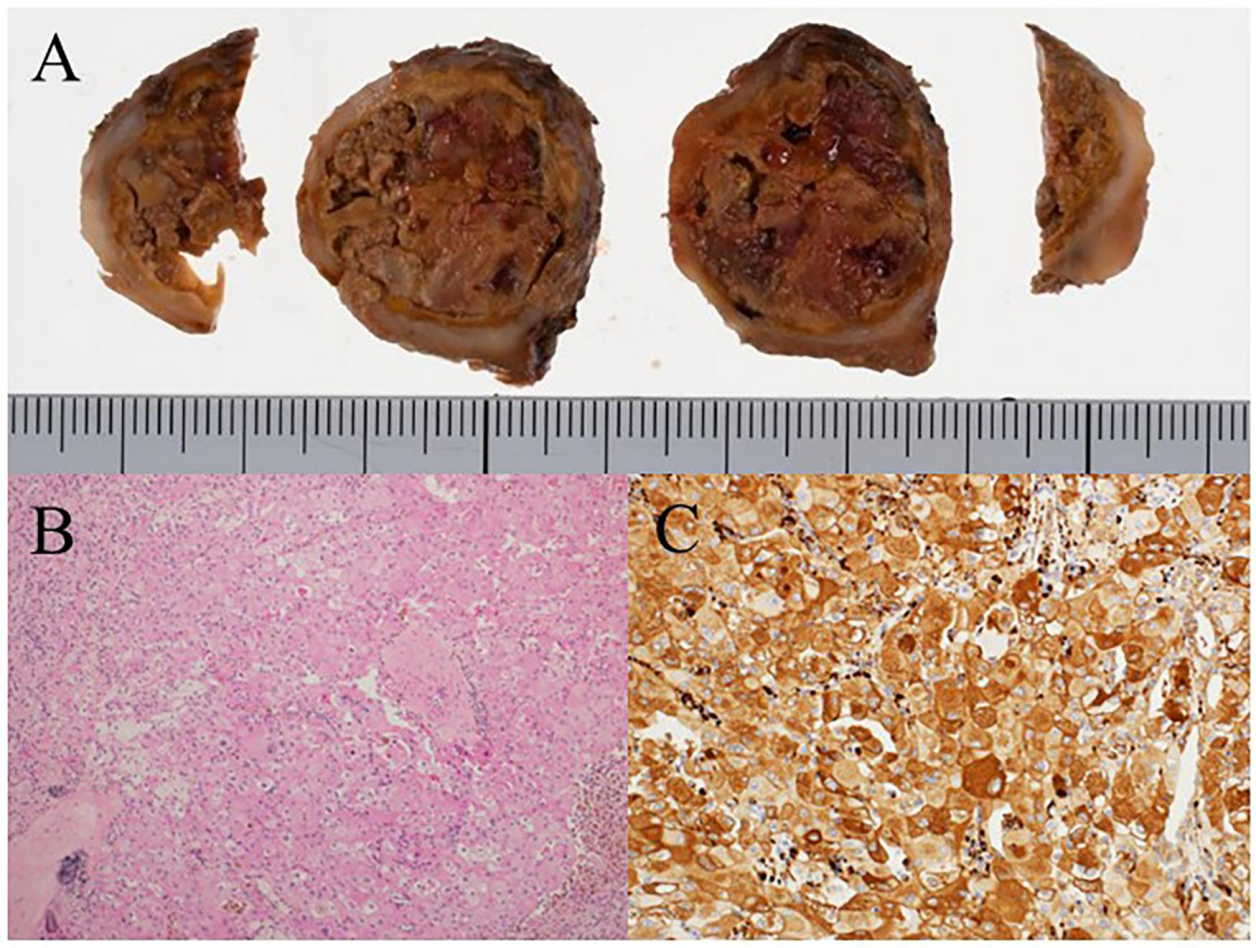
Gross and histopathological findings of the renal tumor. **(A)** Gross ﬁndings of the surgical specimen showing expansive tumor growth and necrosis. **(B)** Microscopic view of the specimen showing polygonal tumor cells with large nuclei and abundant eosinophilic cytoplasm. **(C)** Immunohistochemistry of the tumor showing tumor cell positivity for cathepsin K.

The resected specimen revealed a 35 × 30 × 25 mm solid mass with central necrosis (Figure 2A). Histologically, the tumor consisted of sheets of polygonal cells with plump eosinophilic cytoplasm and bizarre nuclei (Figure 2B). Immunohistochemically, the tumor showed positivity for cathepsin K (Figure 2C), partial positivity for CD10 and p53, and negativity for cytokeratin (AE-1/AE-3), epithelial membrane antigen (EMA), paired box gene 8 (PAX8), S100 protein (S100), human melanoma black 45 (HMB45), melan antigen (Melan A), muscle actin (HHF-35), alpha-smooth muscle actin (α-SMA), and transcription factor E3 (TFE3). On the basis of the collective histological and immunohistochemical features, the tumor was diagnosed as an eAML.

Ophthalmological examination revealed multiple bilateral retinal hamartomas, and brain MRI revealed cortical tubers, whereas head intracranial calcifications were detected using CT. On the basis of these findings, the patient was clinically diagnosed with TSC with polycystic kidneys.

**Figure 3:**
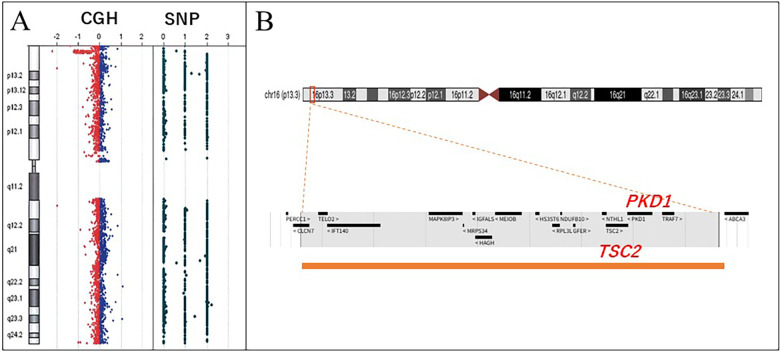
Chromosomal microarray. **(A)** Heterozygous deletion in 16p13.3. **(B)** Deleted area (red square), including an interstitial 882-kb deletion at 16p13.3. Lower panel: enlargement of the deleted region (orange bar). This region includes several genes, including *TSC* and *PKD1*. *TSC*, tuberous sclerosis complex; *PKD1*, polycystin 1.

Although targeted sequencing of *TSC1* and *TSC2* revealed no pathogenic variants of either gene, chromosomal microarray analysis (CMA) identified an 882-kb deletion [arr 16p13.3(1459503_2263638) × 1] in the 16p13.3 region encompassing both *TSC2* and *PKD1* (Figure 3), on the basis of which, the patient was diagnosed with PKDTS.

Postoperatively, the patient's course was uneventful without perioperative complications. Serum creatinine and estimated GFR remained within age-appropriate ranges. Follow-up imaging (renal ultrasound/CT) showed no residual or recurrent mass. At 24 months, there was no evidence of recurrence, and renal function remained stable under routine surveillance. Written informed consent for publication of this case, including clinical details and images, was obtained from both parents, and assent was obtained from the patient. Ethics committee approval was waived in accordance with the institutional policy.

## 3 Discussion

This represents the pediatric case of PKDTS presenting with AML rupture at age 13, manifesting as acute abdominal pain. TSC typically involves angiomyolipoma, multiple renal cysts, and, less frequently, RCC; these points have been outlined above and in the Introduction. Among patients with TSC, the prevalence of AML is significantly higher in those with *TSC2* variants (59.2%) than in those with variants of *TSC1* (33.3%). Furthermore, patients with *TSC2* variants tend to be younger and experience more rapid tumor growth, although a majority of AMLs remain clinically asymptomatic (4). Although comparative studies of *TSC1-* and *TSC2*-related disease are relatively limited, rapid RCC enlargement has been reported in patients with TSC (8), and given that renal lesions in TSC carry the risk of spontaneous rupture, regular follow-up and careful management are required having established a clinical diagnosis (6, 9).

In the present case, ongoing follow-up was interrupted on account of the patient's anxiety regarding medical visits, resulting in a delayed diagnosis of TSC until rupture of the renal lesion prompted further medical evaluation. Such cases of incidental TSC diagnosis following rupture of a renal mass appear to be exceedingly rare; to the best of our knowledge, no prior pediatric reports have explicitly described PKDTS in this context. In our experience, when TSC is suspected, early clarification of the diagnostic plan promotes timely diagnosis and may prevent later complications. Clear follow-up instructions and access to psychological support may help mitigate anxiety-related loss to follow-up.

Given the presence of multiple renal cysts, we performed comprehensive genetic evaluation with a consideration of PKDTS. PKDTS results from a contiguous deletion involving TSC2 and PKD1 and accounts for ∼2%–5% of T SC. It is characterized by early-onset, rapidly progressive bilateral cystic kidney disease with enlarged kidneys and a higher renal tumor burden, and it often progresses to end-stage renal disease in adolescence/early adulthood (10, 11). Accordingly, early recognition and rigorous longitudinal surveillance are warranted. Although precise estimates for the prevalence of PKDTS among patients with TSC and polycystic kidneys have yet to be obtained, the syndrome has previously been reported in 22 of 27 (82%) cases, indicating a potentially high frequency (12). Consequently, PKDTS should be strongly suspected in patients with TSC-like clinical features and multiple renal cysts.

In cases clinically diagnosed with TSC, gene-targeted sequencing of *TSC1 or TSC2* is typically performed first in routine practice. However, when atypical features such as ARPKD-like renal cysts are present, PKDTS should be suspected, prompting CMA. This approach is critical because standard targeted NGS panels, optimized for SNVs/indels, frequently miss multi-exonic or whole-gene deletions, including the TSC2 and PKD1 deletion identified by CMA in our patient. Large TSC2 deletions account for ∼6% of pathogenic variants (13), underscoring the need for copy-number analysis when clinical suspicion persists despite negative sequencing.

eAML is a rare histological subtype of AML, data on the prevalence of which is currently limited. Among the few relevant studies that have been conducted, it has been reported that approximately 7% of TSC-associated AMLs are eAMLs (14). In contrast, although TSC is present in approximately 10% of AML cases, approximately 50% of eAML cases occur in association with TSC, which accordingly tends to indicate that eAML is a renal neoplasm relatively specific to TSC (15).

Whereas TSC-associated RCC is a distinct oncological entity with specific clinical and histopathologic features, unlike AML, it is currently not among the formal diagnostic criteria for TSC, and eAML is considered a histological variant of AML rather than a distinct entity (16, 17). However, although the “second-hit” mechanism in TSC and its potential contribution to RCC development are well-established, a direct pathogenic link with eAML remains to be determined (18).

Large eAMLs, particularly those exceeding 7 cm in diameter, have been reported to undergo distant metastases in approximately one-third of cases, thereby highlighting the need for stringent clinical surveillance (19). Conversely, other studies have reported a very low metastatic potential, and, consequently, there is currently a lack of consensus regarding the malignant nature of eAML (20, 21). Although no standardized treatment guidelines have been established, complete surgical excision is considered desirable at the time of diagnosis in cases, such as that reported herein, in which it is difficult to differentiate eAML from RCC. After resolution of rupture-related inflammation, we chose right partial nephrectomy rather than conservative management or transarterial embolization. Calcification on CT is atypical for AML and raised concern for RCC or eAML, making tissue diagnosis decisive for subsequent care. Given the patient's age and underlying cystic kidneys, embolization would not address the diagnostic uncertainty and carries a risk of incomplete control and recurrence, whereas robot-assisted partial nephrectomy provided definitive histology while preserving renal parenchyma. This strategy aligns with our differential diagnosis and the need to balance oncologic safety with long-term renal function. Two year after surgery, the patient's renal function had remained stable without any evident deterioration.

The pathological differential diagnosis of eAML from other renal tumors requires careful evaluation. The main differential diagnoses include RCC and malignant melanoma. In the present case, negative immunohistochemical staining for cytokeratin (AE-1/AE-3), EMA, and PAX8 served to exclude the likelihood of RCC. Moreover, although cathepsin K positivity can be a feature of TFE3-rearranged RCC, malignant melanoma, and eAML, the absence of TFE3 immunoreactivity ruled out TFE3-rearranged RCC. Similarly, negative staining for S100, HMB45, and Melan A excluded malignant melanoma. HMB45-positive tumor cells in eAMLs generally show a scattered appearance, whereas cathepsin K show diffuse strong positivity. In the present case, negativity of HMB45 may be due to the degeneration and necrosis of the tumor cells. Collectively, these immunohistochemical findings tended to be consistent with a diagnosis of eAML (22, 23). The immunohistochemical panel may be diagnostically helpful for eAML.

Although extremely rare in children, eAML has been reported in individuals as young as 4 years of age, and, consequently, should not be excluded on the basis of age alone (24). Similarly, whereas in patients with TSC, when RCC develops, this typically occurs at a mean age of approximately 36 years, it can present as early as 2 years of age, and hence young age alone should not rule out this condition (25, 26). AMLs often enlarge during puberty, which is an important consideration for clinical management. These tumors are also more commonly detected in females, and rapid enlargement or rupture during pregnancy has been reported, which is speculated to be associated with a promotion of tumor cell proliferation by estrogen and progesterone (27, 28). In males, androgens, such as testosterone, may also indirectly contribute to tumor growth. Similarly, in RCC, the expression of sex steroid hormone receptors is implicated in tumor progression and prognosis (29). However, although RCC is generally asymptomatic, rapid enlargement during adolescence, with abdominal pain as the initial symptom, has been documented, resulting in a clinical course similar to that observed in the present case (8). Conversely, calcification and necrosis on imaging are atypical features associated with AML and can serve as valuable diagnostic clues (30). Consequently, tumor biopsies should be considered to establish an accurate differential diagnosis in pediatric patients with TSC showing renal lesions atypical of AML on imaging analyses using modalities such as CT or MRI.

This report has several limitations. First, it describes a single pediatric case, which limits generalizability. Second, the follow-up period is relatively short, and longitudinal renal imaging prior to rupture was unavailable, precluding assessment of pre-rupture growth kinetics. Third, causal inferences regarding the relationship between PKDTS and the behavior of eAML cannot be drawn from this study design.

In conclusion, this case represents an extremely rare instance in which rupture of an eAML led to an incidental diagnosis of PKDTS. However, as similar cases may be encountered in the future, clinicians should accordingly be aware of this diagnostic possibility. Moreover, the fact that neither eAML nor RCC can be excluded, even in pediatric patients, is an important consideration for the management of such patients.

Although radiological differentiation between these entities remains challenging, the presence of calcification or necrosis, which are atypical features of AML, should prompt active consideration of renal biopsy to establish an accurate diagnosis.

## Data Availability

The original contributions presented in the study are included in the article/Supplementary Material, further inquiries can be directed to the corresponding author.
